# Statewide dissemination and implementation of physical activity standards in afterschool programs: two-year results

**DOI:** 10.1186/s12889-018-5737-6

**Published:** 2018-07-03

**Authors:** Michael W. Beets, R. Glenn Weaver, Keith Brazendale, Gabrielle Turner-McGrievy, Ruth P. Saunders, Justin B. Moore, Collin Webster, Mahmud Khan, Aaron Beighle

**Affiliations:** 1 Arnold School of Public Health, University of South Carolina, 921 Assembly St, 1st Flr Suite, RM 131, Columbia, SC 29208 USA; 2 Wake Forest School of Medicine, Wake Forest University, Winston-Salem, NC USA; 3 University of Kentucky, Lexington, KY USA

## Abstract

**Background:**

In 2015, YMCA afterschool programs (ASPs) across South Carolina, USA pledged to achieve the YMCA physical activity standard calling for all children to accumulate 30 min of moderate-to-vigorous physical activity (MVPA) while attending their ASPs. This study presents the final two-year outcomes from the dissemination and implementation efforts associated with achieving this MVPA standard.

**Methods:**

Twenty ASPs were sampled from all South Carolina YMCA-operated ASPs (*N* = 97) and visited at baseline (2015) and first (2016) and second year (2017) follow-up. All ASPs were provided training to increase MVPA during the program by extending the scheduled time for activity opportunities and modifying commonly played games to increase MVPA. The RE-AIM framework was used to evaluate the statewide intervention. Accelerometer-derived MVPA was the primary outcome. Intent-to-treat (ITT) models were conducted summer 2017. Programs were also classified, based on changes in MVPA from 2015 to 2016 and 2016–2017, into one of three categories: gain, maintain, or lost. Implementation, within the three groups, was evaluated via direct observation and document review.

**Results:**

Adoption during the first year was 45% of staff attending training, with this increasing to 67% of staff during the second year. ITT models indicated no increase in the odds of accumulating 30 min of MVPA after the first year for either boys (odds ratio [OR] 1.06, 95CI 0.86–1.31) or girls (OR 1.14, 95CI 0.87–1.50), whereas an increase in the odds was observed during the second year for boys (OR 1.31, 95CI 1.04–1.64) and girls (OR 1.50 95CI 1.01–1.80). Programs that lost MVPA (avg. − 5 to − 7.5 min/d MVPA) elected to modify their program in a greater number of non-supportive ways (e.g., reduce time for activity opportunities, less time spent outdoors), whereas ASPs that gained MVPA (avg. + 5.5 to + 10.1 min MVPA) elected to modify their program in more supportive ways.

**Conclusions:**

The statewide study demonstrated minimal improvements in overall MVPA. However, child MVPA was dramatically influenced by ASPs who elected to modify their daily program in more supportive than non-supportive ways, with no one program modifying their program consistently across the multi-year initiative. These findings have important implications for organizations seeking to achieve the MVPA standard.

**Trial registration:**

Clinical Trial Registration: NCT02394717.

## Background

In 2011, the YMCA of the USA adopted the Healthy Eating and Physical Activity Standards (HEPA) for all their afterschool programs (ASPs) [1]. The standard for physical activity called upon ASPs to ensure each child accumulates a minimum of 30 min of moderate-to-vigorous physical activity (MVPA) each day during the ASP. The achievement of the MVPA standard holds considerable public health importance as it would ensure children accumulate at least half of their daily MVPA recommendation during the ASP. Although the MVPA standard was adopted in 2011, data collected during the spring of 2015 on children’s activity levels in YMCA-operated ASPs demonstrated that only 33% of boys and 17% of girls were achieving the standard [2]. This is consistent with other published evaluations of non-YMCA operated ASPs [3–5].

Given the adoption of the HEPA standards in 2011 was insufficient to improve routine practice within ASPs to achieve the 30 min MVPA standard, the YMCAs across a single southeastern state in the United States, South Carolina (SC), pledged to achieve the MVPA Standard using a coordinated statewide training framework to enhance the quality of their programming to promote greater amounts of physical activity. The initiative focused on providing professional development training for program staff, as well as modifying program structure to facilitate opportunities for children to engage in physical activity. The training built upon previous studies by focusing on enhancing the quality of existing physical activity opportunities and extending the amount of time allocated for physical activity [6–11]. Results from the first year (2015 baseline to 2016 first year) indicated no overall changes in the proportion of children accumulating 30 min MVPA [12]. However, this was greatly influenced by whether ASPs attended the trainings and what components, if any, the ASPs implemented. Based on the first year outcomes, the CEOs of each SC YMCA Association pledge to have all their ASP staff receive the professional development training for 2017. The objective of this study is to report the two year outcomes (2015 to 2017) from this statewide initiative.

## Methods

The methodology and intervention for this study is described in detail in previous publications [2, 12] and will be overviewed in brief below. For this study, baseline data were collected during the spring of 2015. The delivery of the intervention occurred during the fall of 2015 and fall 2016, while outcomes following the trainings were measured during the spring 2016 and spring 2017. This study employed a single group quasi-experimental pre/post design, with all YMCA-operated ASPs in South Carolina eligible to participate in the professional development trainings. The study is reported in accordance with the Transparent Reporting of Evaluations with Nonrandomized Designs (TREND) statement [13]. Additionally, the study findings are reported using the RE-AIM framework (reach, effectiveness, adoption, implementation, and maintenance) [14, 15]. The maintenance component of the framework was not assessed given the absence of a post-treatment follow-up (e.g., > 6 months) [15]. Also, reach and adoption were considered analogous in this study given the intervention targeted change at the setting-level (i.e., ASPs) that was hypothesized to lead to changes in the MVPA levels of the children attending (i.e., individuals enrolled in the ASPs) [15, 16].

### Sampling strategy for evaluation of ASPs

A detailed description of the sampling strategy for the evaluation ASPs is reported elsewhere [2]. In brief, the sampling strategy included a single program from each of the 20 Associations. For Associations that operated a single program (*n* = 5), that program was selected. For Associations that operated two or more programs (*n* = 15), the following sampling strategy was employed. For Associations where all programs enrolled fewer than 50 children (*n* = 3), the largest program was selected. For Associations that operated programs with more than 50 children enrolled (*n* = 12), a single program was randomly selected from these. All sites selected as an evaluation site, both randomly and non-randomly, agreed to participate as part of their Association’s statewide commitment to the initiative. All parents were informed of the study from their respective ASP location. Parents were provided an option to opt-out (passive consent) their child from participating. Children verbally assented on each day where data occurred to participate in the measures. All methods were approved by the Institution Review Board of the University of South Carolina.

### Child and ASP characteristics

Child demographics were self-reported (e.g., age, grade, race/ethnicity), and standing height and weight were measured using standard protocols with children removing shoes and wearing light clothing [17]. The annual revenue for each YMCA Association was collected from their most recent publicly available annual reports from 2013. The percentage of households in poverty was used as a marker of socioeconomic status of the ASPs. This was based on US Census 2014 zip code information based on the operating location zip code for each ASP. Each ASP’s operating setting, either in a school or YMCA facility, was also recorded.

### Intervention description

The intervention for physical activity followed the Strategies To Enhance Practice (STEPs) framework [18–22]. All intervention-related activities were coordinated and delivered by a single full-time employee with 15 years of experience operating YMCA youth programs. For the statewide initiative, the STEPs training was divided into two distinct, yet complimentary, trainings. The two different types of trainings were offered over the course of the fall academic year (August to October 2015 and 2016). The first training was for site leaders (i.e., the individuals directly responsible for a single ASP). The second was for site leaders and staff (i.e., those individuals responsible for interacting and overseeing children during the program, referred to as the all-staff training). For the first year, attendance at the trainings was voluntary, with ASPs opting to attend both, one, or neither of the trainings. For year two, the CEOs of the YMCA Associations pledged to have all their staff attend the trainings.

The site leader training lasted for two hours and provided an overview and history of the YMCA MVPA standard and strategies to increase children’s MVPA in ASPs. Strategies were based on the Theory of Expanded, Extended, and Enhanced Opportunities [22] which focused on extending the amount of allocated time for children to be physically active each day, creating schedules that clearly define the roles and responsibilities of staff during physical activity (PA) opportunities and other scheduled times, and enhancing the games commonly played in the ASPs by using the LET US Play principles (lines, elimination, team size, uninvolved staff/kids, and space, equipment and rules) for modifying games to maximize MVPA [18, 19, 23]. Programs were also asked that when time was scheduled for physical activity, that children did not have a choice of selecting a non-active alternative (e.g., play on playground or stay inside at computer lab). For allocated PA time, where ASPs were allocating 50% (i.e., 90mins) or more of their daily schedule for PA opportunities, they were asked to maintain this allotment. This was based on information collected from the baseline evaluation of ASPs in 2015, where ASPs scheduled a median of 59% (~115mins/d) of their daily program for PA opportunities, with 15 ASPs devoting 50% or more of their daily schedule for PA. For any ASP that scheduled less than 60 min for PA, they were asked to increase this to a minimum of 60 min per day (33% of a 3 h program). During the first year a total of three site leader trainings were provided, one per region in the state (i.e., Low Country, Midlands, and Up State). For the second year, no separate site leader only trainings were provided. This was based on the low attendance during the first year (only 16% attended). The content of the site leader trainings was, therefore, incorporated into the all-staff trainings and held for two hours prior to the start of the all-staff trainings. This eliminated the need for site leaders to schedule to attend a training on two separate days.

The second training, the all-staff training, lasted for two hours and provided an overview of the YMCA MVPA standard and strategies to increase MVPA in ASPs. Strategies included role modeling PA behaviors, scheduling time for children to be active, and following the LET US Play principles. The majority of the training focused on skill development for using the LET US Play principles. Attendees identified a game they commonly play, played the game using its traditional rules, and then played the game again with self-identified modifications based on the LET US Play principles. This procedure was repeated for up to five different games. Throughout the training, the LET US Play principles were continuously introduced, explained, demonstrated, and reinforced. For the first year, a total of 13 all-staff trainings were conducted. For the second year, a total of 16 all-staff trainings were conducted, with four Associations attending the same regional training. Additionally, monthly emails were distributed to all ASP site leaders. Emails included STEPs-related physical activity promotion content, such as examples of LET US Play principles, links to online two-minute videos depicting a LET US Play principle, and scheduling physical activity opportunities.

### Reach/adoption, effectiveness, and implementation of STEPs

All measurements occurred during the spring (March through April) of 2015, 2016, 2017. Consistent with previously established protocols, each ASP was visited for data collection on four non-consecutive, unannounced days Monday through Thursday [5, 24–26].

#### Reach/adoption

For the purpose of this study, the measure of the reach/adoption of the intervention was determined by percentage of Associations, ASPs, site leaders and staff in attendance at the fall trainings for professional development at each of the intervention years. To document reach/adoption, at each training offered during the fall of 2016 and 2017 attendees provided their name, position, and their affiliated ASP and Association name.

#### Effectiveness

The effectiveness of the STEPs intervention was evaluated via accelerometry using a standardized protocol as the primary outcome [2, 12]. All children attending an ASP on measurement days were fitted with an ActiGraph GT3X+ accelerometer on the hip. Accelerometer data were distilled using 5-s epochs to account for the intermittent and sporadic nature of children’s PA [27] and to capture the transitory PA patterns of children [28, 29]. Upon arrival to the ASP, children were fitted with an accelerometer and the arrival time was recorded (monitor time on). Research staff continuously monitored the ASP for accelerometer wear compliance. At the time of a child’s departure, research staff removed the accelerometer and recorded the time (monitor time off). Children wore the monitors for the entire time in attendance at the ASPs. Cutpoint thresholds associated with moderate and vigorous activity were used to distill the PA intensity levels [30] and sedentary behavior [31]. Children were included in the study if they had one or more valid days of accelerometer data defined by a total wear time (time off minus time on) of ≥60 min [5, 25, 32]. The minutes all children spent in MVPA were dichotomized to represent those children who achieved (i.e., ≥30 min MVPA/day) and those that failed to achieve (i.e., < 30 min MVPA/day) the PA standard [33].

#### Implementation

Implementation of STEPs was measured via document review and on-site direct observation during all measurement occasions [34–37]. The amount of scheduled PA opportunities was determined via program schedules. Program schedules were collected on each of the four data collection days at each measurement occasion and the time allotted for PA opportunities was totaled for each day. We also measured the PA opportunities via direct observation using the System for Observing Staff Promotion of Activity and Nutrition (SOSPAN) [37]. The observational protocol consisted of continuous systematic scanning and rotating through pre-defined target areas across the entire duration of an ASP. Trained observers completed standardized reliability training and conducted all observations. Inter-rater agreement criteria were set at > 80% using interval-by-interval agreement for each category [37]. Consistent with published reliability protocols [37], reliability was collected prior to measurement and on at least 30% of data collection days. Inter-observer reliability for the LET US Play principles were estimated via interval-by-interval percent agreement and weighted kappa (κw). Percent agreement ranged from 90.5 to 99.2% and κw ranged from 0.21 to 0.96 (median 0.75). Reliability was checked weekly to identify disagreements. Operational definitions of variables with borderline or low reliability (< 90% agreement) were then discussed with observers to ensure reliability and prevent observer drift. A total of 22,626 scans were collected across 2015, 2016, and 2017 data collection periods.

Each scan was tagged with a “context” variable that identified whether the scan occurred during snack, academics, enrichment, physical activity, or some other scheduled ASP opportunity (e.g., water-breaks, transitions). The percentage of scans each day a context variable occurred was also used to assess time allocated for a given context. The percent of scans was compared to the written ASP schedule to determine consistency among the two. Where discrepancies occurred, the direct observation allocated time was used as an indicator of time allocated for a given context. Also, each scan was tagged with a location variable and this was used to calculate time spent outdoors. SOSPAN was used to evaluate the implementation of 12 LET US Play principles during PA opportunities. On each day of observation, the percentage of scans were computed for the following: children waiting in line for turn, children eliminated, small sided-games, staff actively engaged in activity, staff verbally encouraging activity, choice of two or more activity offered, girl’s provided with their own physical activities, staff giving instructions on how to play games, children waiting for activity to start (i.e., idle time), staff withholding PA as punishment, and staff disciplining children with PA. A twelfth variable was collected at the end of each day - all staff wear clothing to be physical active (yes/no). The distribution of each of the first eleven LET US Play principles were divided into tertiles based on their 33rd and 66th percentile for the principle at baseline [36]. Programs were assigned a zero (<33rd centile), 1 (33rd to 66th centile), or 2 (>66th centile). Staff wearing appropriate clothing was transformed into a 3-pt scale ranging from zero (none of the days), 1 (some of the days), or 2 (all days). Scores were summed to represent an overall LET US Play implementation score that could range from 0 to 24.

### Overall implementation and changes in MVPA

First, ASPs were classified into one of three categories based upon their change in MVPA between adjacent years (2016 minus 2015 and 2017 minus 2016) for boys and girls, separately. Change in MVPA was benchmarked at ±3 min MVPA, which is consistent with the most recent meta-analysis on physical activity interventions using objective measures in youth [38]. Second, using the SOSPAN and document review, the following six implementation indicators were created and expressed as a percentage: LET US Play implementation score, amount of PA time observed, amount of PA time scheduled that was PA only time, the total amount of PA time scheduled as documented in daily schedules, and the amount of time the ASPs went outside during scheduled PA opportunities and across the entire duration of the program. For each evaluation ASP, changes in the implementation indicators were calculated between adjacent years. Programs were the classified as having either decreasing (<− 10%), increasing (> + 10%) or no change (> − 9.99 to <+ 9.99%) in level of implementation on each indicator separately. In absence of established benchmarks, change was defined as a ± 10% difference between adjacent years.

### Statistical analyses

Analyses were conducted summer 2017. Descriptive statistics were computed by gender for the percentage of youth meeting the 30 min/day MVPA standard and in mins/d of MVPA. The overall test for the effectiveness of the intervention was examined using intent-to-treat (defined as all ASPs received the intervention) repeated-measures random effects logit models, with days measured nested within children, who were nested within ASPs. The models were estimated using the dichotomized MVPA variable (0 = < 30 min/d) as the dependent variable for boys and girls, separately, to compare changes in the percentage of children meeting the MVPA Standard over time. Race (African Americans), age (years), ASP operating in YMCA facility, and Census 2010 zip code percentage of households in poverty were included in the models as covariates. As a secondary outcome, the minutes spent in MVPA were estimated with random effects quantile regression models at the 50th quantile of the distribution and design-matrix bootstrapped standard errors, separately [39]. The same covariates were used in this model. All analyses were performed using STATA (v.14.0 College Station, TX).

## Results

The descriptive characteristics of the evaluation ASPs and the children enrolled in the programs during spring 2015 (baseline), spring 2016 (end of first year of intervention), and spring 2017 (end of second year of intervention) are presented in Table 1. The reach/adoption of the trainings for the evaluation sites and all YMCA-operated ASPs across the state at the site leader and all staff training occurring fall 2016 and fall 2017 are presented in Table 2. Overall, the attendance at both the site leader and all-staff trainings during the second year were higher than those observed during the first year of the intervention, with 77% of site leaders attending the site leader trainings in year two (versus 16% in year one) and 67% of the staff attending the all-staff training during year two (versus only 45% during year one).

**Table 1:** Baseline (spring 2015), year 1 (spring 2016), and year 2 (spring 2017) characteristics of the afterschool programs and children in attendance

	Baseline (Spring 2015)	End of 1st year (Spring 2016)	End of 2nd year (Spring 2017)
Child Characteristics			
Sample Size (N)	1117	1173	1228
Boys (percentage)	56%	53%	52%
Age (years, SD)	7.7 ± 1.7	8.0 ± 1.7	7.8 ± 1.7
Race/Ethnicity (percentage)			
Black	31%	36%	37%
Hispanic	3%	2%	2%
Other	5%	3%	5%
White	62%	60%	56%
Height (cm, SD)	128.5 ± 11.3	131.8 ± 12.2	130.4 ± 38.1
Weight (lbs, SD)	67.1 ± 23.3	72.0 ± 24.4	73.1 ± 30.6
Afterschool Program Schedule			
Total Program Length (mins/day, SD)	195 ± 29	193 ± 32	200 ± 18
Snack	11% ± 5%	11% ± 5%	11% ± 5%
Academics/Enrichment	26% ± 11%	37% ± 12%	33% ± 9%
Other	6% ± 4%	5% ± 4%	11% ± 8%
Physical Activity	57% ± 16%	47% ± 16%	45% ± 13%
Free Play	58% ± 26%	64% ± 26%	65% ± 29%
Organized	43% ± 26%	36% ± 26%	35% ± 29%
Afterschool Program Characteristics			
Households in Poverty (percent, SD)	13% ± 6		
Receive State of Federal Reimbursement for Snack	35%		
Serve a Hot Meal (percent of ASPs)	10%		
Receive State of Federal Reimbursement for Meal	10%		
Available Program Space (ft^2^, SD)			
Indoor	9128 ± 4386		
Outdoor	137,755 ± 87,095		
Location			
YMCA	44%		
School	56%		
Accelerometer Estimates of Physical Activity			
Sample with valid accelerometer data (N)	1078	1160	1166
Activity Intensity (mins/day, SD)			
Sedentary	64.3 ± 24.6	60.1 ± 22.6	55.8 ± 19.9
Light Physical Activity	41.2 ± 17.9	43.6 ± 15.1	43.0 ± 13.7
Moderate Physical Activity	11.0 ± 6.6	11.6 ± 6.1	11.8 ± 5.3
Vigorous Physical Activity	10.4 ± 7.7	10.7 ± 7.2	12.0 ± 7.3
Moderate-to-Vigorous Physical Activity	21.4 ± 13.5	22.3 ± 12.4	23.8 ± 11.8
Percent Meeting MVPA 30 min/day Standard	25.9%	26.5%	30.0%
Total Time in Attendance	127.0 ± 34.2	126.0 ± 33.5	122.6 ± 30.0

Percentages may not add to 100% due to rounding

**Table 2:** Attendance at the professional development trainings offered year 1 fall 2016 and year 2 fall 2017

	Fall site leader training			Fall all-staff training					
	2 h training covering scheduling and overview of LET US Play principles			2 h training covering physical activity role modeling and LET US Play activity modifications					
	Site leader			Site leader			Staff		
	Actual	Attended	%	Actual	Attended	%	Actual	Attended	%
Year 1^a^									
Overall - Number Individuals	116	19	16%	116	75	65%	474	211	45%
Number of Associations	20	12	60%	20	14	70%	20	12	60%
Year 2^b^									
Overall - Number Individuals	111	86	77%	111	85	77%	535	356	67%
Number of Associations	20	19	95%	20	19	95%	20	19	95%

^a^Year 1, Site Leader training occurred on a separate day than the All-Staff training and were provided on 3 different occasions

^b^Year 2, Site Leader training occurred prior to All-Staff training on same day

**Fig. 1:**
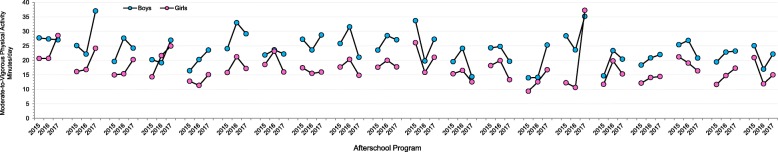
Changes in moderate-to-vigorous physical activity minutes per day for boys and girls by afterschool program from 2015, 2016, and 2017

For effectiveness, the change in the proportion of boys and girls achieving the 30 min/d MVP Standard and the minutes/d of MVPA accumulated from across all three measurement occasions are presented in Table 3 and changes in mins/d MVPA for each ASP for boys and girls separately are presented in Fig. 1. Overall, from 2015 to 2017, there was a statistically significant increase in the odds of both boys and girls achieving the 30 min/d MVPA Standard by 1.31 (95CI 1.04–1.64) and 1.35 (95CI 1.01–1.80), respectively. This represented an increase of 5 and 3% for boys and girls achieving the MVPA Standard, respectively. Correspondingly, during the last year of the intervention, boys increased the number of mins/d of MVPA by 2.6 (95CI 1.0–4.1) and girls by 1.5 (95CI 0.1–2.9).

**Table 3:** Estimates and changes in accelerometer-derived moderate-to-vigorous physical activity (MVPA) from 2015, 2016, and 2017

Physical activity estimate	Year	Girls		Boys	
		M	SD	M	SD
Moderate-to-Vigorous Physical Activity (minutes/day)^a^	2015	18.2	±11.3	24.2	±14.7
	2016^c^	19.0	±10.2	25.3	±13.4
	2017^d^	20.1	±11.8	27.1	±14.1
		Est (95CI)		Est (95CI)	
Change in MVPA Minutes/day^b^	2016^c^	1.4 (−0.1, 2.9)		0.9 (−0.6, 2.5)	
	2017^d^	**1.5 (0.1, 2.9)**		**2.6 (1.0, 4.1)**	
		%		%	
Prevalence of Children Achieving 30 min/d MVPA^a^	2015	17.3		33.5	
	2016^c^	17.7		34.6	
	2017^d^	20.3		38.8	
		OR (95CI)		OR (95CI)	
Odds of Accumulating 30 min/d MVPA^b^	2016^c^	1.14 (0.87, 1.50)		1.06 (0.86, 1.31)	
	2017^d^	**1.35 (1.01, 1.80)**		**1.31 (1.04, 1.64)**	

**BOLDED** values are statistically significant at *P* < 0.05

^a^Unadjusted values

^b^Model implied estimates adjusting for race (African Americans), age (years), ASP operating in YMCA facility, and Census 2010 zip code percentage of households in poverty

^c^First year of intervention delivery and implementation compared to baseline

^d^Second year of intervention delivery and implementation compared to baseline

For implementation, changes across the implementation indicators by ASPs that either gained (+3mni/d), lost (− 3 min/d), or maintained MVPA across each adjacent measurement occasion (2015 vs 2016 and 2016 vs 2017) are presented in Tables 4 and 5. For boys, 7 of the possible 9 patterns of gain-lost-maintained classifications were observed, with only 2 ASPs classified as maintain-maintain and only a single ASP exhibiting gains across both years (i.e., gain-gain). Similar findings were found for girls, with 8 of the 9 patterns observed, with only 3 ASPs classified as maintain-maintain and 1 classified as gain-gain. Importantly, the gain-gain ASP for boys was not the same gain-gain ASP for girls. Across adjacent years, and for both boys and girls, ASPs that gained MVPA had a consistently higher supportive to non-supportive change ratio (range 1.0 to 2.0) in the implementation indictors compared to ASPs classified as maintain (range 0.6 to 0.8), followed by ASPs classified as lost (range 0.1 to 0.4).

**Table 4:** Levels of implementation by gain, maintain, or lost minutes of moderate-to-vigorous physical activity for boys

	2015 to 2016								2016 to 2017							
				Percent ASP Δ ±10%		Avg No. Δ		Ratio supportive to non-supportive				Percent ASP Δ ±10%		Avg No. Δ		Ratio supportive to non-supportive
	2015	2016	Avg. Δ	% Decreased	% Increased	Supportive	Non-supportive		2016	2017	Avg. Δ	% Decreased	% Increased	Supportive	Non-supportive	
Gain																
# ASPs			9								8					
LET US Play Index^a^	59%	53%	−5%	33%	11%	1.6	1.6	1.0	49%	52%	2%	0%	11%	2.0	1.0	2.0
Observed PA Time^a^	56%	50%	-5%	44%	22%				39%	54%	15%	0%	67%			
PA Only Time^b^	48%	30%	− 10%	33%	11%				20%	40%	21%	22%	56%			
PA Time spent Outdoors^a^	58%	74%	16%	22%	78%				57%	44%	−13%	44%	0%			
Total ASP Time Outdoors^a^	39%	46%	9%	11%	22%				28%	25%	−3%	0%	0%			
Total time PA scheduled^b^	107.1	98.6	− 5.0	33%	22%				83.8	86.9	3.1	22%	44%			
MVPA^c^	23.3	29.6	6.2						21.1	31.2	10.1					
Maintain																
# ASPs			6								7					
LET US Play Index^a^	58%	55%	−3%	17%	17%	1.5	2.3	0.6	52%	50%	−2%	14%	0%	1.3	2.0	0.6
Observed PA Time^a^	60%	52%	−8%	50%	17%				46%	44%	−2%	29%	29%			
PA Only Time^b^	37%	24%	− 14%	50%	33%				26%	41%	15%	14%	71%			
PA Time spent Outdoors^a^	75%	85%	10%	17%	33%				72%	58%	−14%	43%	14%			
Total ASP Time Outdoors^a^	47%	49%	2%	17%	33%				36%	29%	−8%	43%	0%			
Total time PA scheduled^b^	102.5	87.5	− 15.0	83%	17%				94.3	78.6	−15.7	57%	14%			
MVPA^c^	23.8	23.9	0.1						27.6	27.2	− 0.4					
Lost																
# ASPs			5								5					
LET US Play Index^a^	50%	51%	1%	0%	20%	1.0	2.4	0.4	57%	50%	−6%	60%	0%	1.0	3.8	0.3
Observed PA Time^a^	59%	34%	− 25%	80%	0%				57%	49%	−9%	60%	20%			
PA Only Time^b^	51%	21%	− 30%	60%	0%				39%	27%	−12%	60%	20%			
PA Time spent Outdoors^a^	43%	41%	− 1%	20%	40%				77%	47%	−30%	80%	20%			
Total ASP Time Outdoors^a^	31%	18%	−13%	40%	0%				60%	28%	−32%	60%	20%			
Total time PA scheduled^b^	98.0	95.0	− 3.0	40%	40%				99.0	75.0	−24.0	60%	20%			
MVPA^c^	29.2	21.7	− 7.5						29.1	21.4	−7.7					

^a^Direct Observation via SOSPAN (System for Observing Staff Promotion of Activity and Nutrition)

^b^Posted Afterschool Program Schedule

^c^Accelerometer

**Table 5:** Levels of implementation by gain, maintain, or lost minutes of moderate-to-vigorous physical activity for girls

	2015 to 2016								2016 to 2017							
				Percent ASP Δ ±10%		Avg No. Δ		Ratio supportive to non-supportive				Percent ASP Δ ±10%		Avg No. Δ		Ratio supportive to non-supportive
	2015	2016	Avg. Δ	% Decreased	% Increased	Supportive	Non-supportive		2016	2017	Avg. Δ	% Decreased	% Increased	Supportive	Non-supportive	
Gain																
# ASPs			6								9					
LET US Play Index^a^	57%	51%	− 6%	50%	17%	2.0	1.9	0.9	49%	51%	1%	11%	11%	1.5	1.4	1.1
Observed PA Time^a^	55%	51%	− 4%	67%	33%				43%	52%	12%	11%	78%			
PA Only Time^b^	50%	40%	− 10%	50%	17%				21%	36%	16%	22%	56%			
PA Time spent Outdoors^a^	54%	77%	23%	17%	83%				60%	36%	−22%	67%	0%			
Total ASP Time Outdoors^a^	33%	48%	15%	0%	33%				28%	19%	−7%	22%	0%			
Total time PA scheduled^b^	106.7	92.5	−14.2	50%	33%				76.4	74.3	−2.2	22%	22%			
MVPA^c^	15.7	21.2	5.5						16.3	25.3	8.0					
Maintain																
# ASPs			12								5					
LET US Play Index^a^	53%	52%	0%	8%	17%	1.4	1.9	0.7	53%	54%	1%	0%	0%	1.4	1.6	0.8
Observed PA Time^a^	56%	42%	−14%	42%	0%				48%	49%	0%	20%	20%			
PA Only Time^b^	38%	15%	− 23%	42%	8%				24%	43%	19%	20%	80%			
PA Time spent Outdoors^a^	56%	63%	8%	25%	50%				75%	52%	−24%	40%	0%			
Total ASP Time Outdoors^a^	36%	34%	− 2%	25%	8%				43%	25%	−18%	40%	0%			
Total time PA scheduled^b^	94.5	84.1	− 10.5	50%	17%				102.0	92.0	−10.0	40%	40%			
MVPA^c^	17.2	17.6	0.3						19.3	18.9	−0.3					
Lost																
# ASPs			2								6					
LET US Play Index^a^	60%	51%	−9%	100%	0%	0.5	3.5	0.1	56%	49%	−7%	33%	0%	1.0	2.8	0.4
Observed PA Time^a^	64%	53%	− 11%	50%	0%				50%	42%	−8%	50%	17%			
PA Only Time^b^	46%	42%	−4%	50%	0%				37%	32%	−5%	50%	33%			
PA Time spent Outdoors^a^	66%	63%	−3%	50%	0%				74%	64%	−11%	50%	17%			
Total ASP Time Outdoors^a^	48%	38%	− 10%	50%	0%				52%	34%	−17%	33%	17%			
Total time PA scheduled^b^	98.3	115.0	16.7	50%	50%				100.0	77.5	−22.5	67%	17%			
MVPA^c^	24.4	16.9	− 7.5						21.9	16.9	−5.0					

^a^Direct Observation via SOSPAN (System for Observing Staff Promotion of Activity and Nutrition)

^b^Posted Afterschool Program Schedule

^c^Accelerometer

## Discussion

This study reports the outcomes from a statewide initiative to achieve the 30 min/d MVPA standard adopted by YMCA-operated afterschool programs after two years of implementation. Overall, across the two-year implementation/dissemination effort, small improvements were observed in the proportion of boys and girls achieving the 30 min/d of MVPA benchmark. This was in spite of an increase in the attendance of both site leaders and staff that received the training in 2017 compared to the percentage that received the training in 2016. Detailed process evaluation revealed there was substantial variability in the uptake and continuance of different intervention strategies within and between afterschool programs, across the years. This is an important finding as level of implementation was associated with considerable increases or decreases in the amount of MVPA children accumulated during the programs. This bidirectional pattern across years demonstrates the complexity of modifying this environment to promote higher levels of MVPA. Yet despite variability in implementation and the associated improvements or decreases in child activity levels, ASPs were routinely affording children meaningful amounts of MVPA (> 15 to 20 min/d MVPA), indicating that these programs, even without fully achieving the MVPA Standard, can have a positive impact on child activity levels.

Important findings from this study are those associated with the implementation of the strategies that targeted modifiable aspects of daily programmatic structure (e.g., increasing the amount of time allocated for physical activity opportunities, staff enhance commonly played games to promote more MVPA), that when changed, should lead to increased child MVPA during the program. Across both intervention years, ASPs elected to improve upon one or more of the targeted intervention strategies. Conversely, ASPs also elected to modify one or more of the targeted intervention strategies in a less supportive way. This led to a scenario where during each year of evaluation all programs were observed making both supportive and non-supportive changes to how their program operated. Further, no two programs made all the same changes to all the same implementation indicators. This complex pattern of changes suggests that programs can, and will, make alterations to their routine operation that fit best with their local conditions and resources, and that no one strategy is ultimately responsible for improvements (or losses) in child MVPA. For example, an ASP could adopt activity supportive practices such as scheduling more time for children to be physically active each day and schedule more of this time to occur outside, yet simultaneously adopt activity non-supportive practices such as decreasing the quality of the activities and the amount of the scheduled activity opportunities where children cannot elect to engage in a sedentary activity (e.g., play outside or choose to go to a computer lab). Conversely, another ASP may reduce allocated time for activity opportunities, yet make all the allotted time ‘PA only’ time (i.e., children cannot select to do a non-active activity).

The patterns observed from these ASPs are consistent with the theoretical tenets of equifinality [40, 41], whereby the same outcome (in this case increases/decreases in MVPA) can be achieved via varying/multiple pathways (in this case different implementation indicators). The results presented in Tables 4 and 5 illustrate this phenomenon, with programs across the three categories of gain, maintain, and lost MVPA showing both supportive and non-supportive changes in the implementation indicators. An important aspect is that ASPs that gained MVPA during any adjacent year comparison elected to make more supportive changes than non-supportive changes compared to those programs that either maintained or lost MVPA. Overwhelmingly, programs classified as lost MVPA consistently made more non-supportive than supportive changes to their daily program which led to an average decrease in MVPA ranging from 5.0 to 7.7 mins/d. Moreover, the majority of ASPs showed variable levels of MVPA across the three years of evaluation, with some of the changes (both supportive and non-supportive) being very large. This suggests that within any two or three year time period, major swings in MVPA may occur and that ASPs voluntarily make both supportive and non-supportive changes to their daily programmatic schedule that influences child levels of MVPA. Unfortunately, the only other evidence of this phenomenon can be found in the authors’ previous studies [20, 21] that report estimates of MVPA across multiple years for each ASP individually. What these data show is that during any two-year comparison, there appears to be natural “ups and downs” within and across these settings – even for ASPs operated by the same organization [20, 21]. Nevertheless, programs looking to increase MVPA should seek to make more overall supportive changes to their programmatic structure. Which changes they should target, however, is unknown, only that more supportive than non-supportive changes need to be made and ASPs need to do this consistently across years.

Statewide commitment by YMCA CEOs to ensure all staff receive training in August 2016 led to higher levels reach/adoption as indicated by the attendance by both site leaders and staff to the professional development trainings compared to the attendance during trainings in August 2015. The reason 100% attendance was not achieved during the second year was due to conflicting schedules of some employees with the dates of the trainings. Despite this, a much higher percentage of site leaders attended the site leader training during 2016 (77%) compared to only 16% across the state in 2015, whereas 67% of staff received training in 2016 compared to 45% in 2015. Unfortunately, this increase in the number of individuals trained did not translate into full achievement of the MVPA standard. Based on the activity levels presented in Table 2, even though no ASP fully met the 30 min/d MVPA standard, all programs afforded children the opportunity to engage in a large volume of MVPA across all measurement years. This amount of MVPA is consistent with other intervention studies [6, 8, 20, 21, 32] within the ASP setting that report children, prior to their ASP receiving a formal intervention, engage in anywhere from 15 to 30 min of MVPA/d. These MVPA levels suggests the ASP setting may not be a place in need of a physical activity-focused intervention and that efforts should be focused on increasing the number of children who can access, and thereby, benefit from attending an ASPs. National estimates indicate ~ 10 million children currently attend an ASP [42], yet an additional 19 million children would attend if one were available [42]. Providing access to ASPs for these children may result in greater public health gains for MVPA compared to the minimal improvements that can be achieved for the limited number of children who currently have access to ASPs.

The current study has several strengths. These include objective process measures that were linked to objective outcome measures, evaluation of a statewide effort to achieve a nationally endorsed policy, a large number of participating ASPs and children, and multiple years of evaluation. Conversely, the limitations include the lack of a control/comparison group, the findings only represent efforts from a single state, and no planned follow-up to monitor maintenance. It should also be noted that the intervention delivered may not be entirely effective and that alternative approaches may be necessary. However, previous studies that have employed pre-packaged curricula [6, 8–10, 43, 44] or dedicated greater amounts of time to professional development [6] have shown no more effectiveness than the current approach. Thus, questions still remain as to the “best” approach for achieving the 30 min/d MVPA standard and, as mentioned above, whether the time and resources dedicated to increase MVPA within this setting are warranted.

## Conclusion

In conclusion, this two-year evaluation found that, overall, minimal gains in MVPA were made. Detailed process evaluation, however, indicated that all ASPs made both supportive and non-supportive changes to their daily programmatic structure which led to increases and decreases in children’s MVPA, with those ASPs electing to make more supportive changes exhibiting the largest levels of MVPA whilst those ASPs electing to make a greater number of non-supportive changes exhibiting the greatest declines in child MVPA. Future studies need to take account for the substantial within and between program variability across years. Furthermore, intervention scientists need to determine whether intervening within this setting, given baseline MVPA levels, is necessary or whether placing resources towards increasing access to these programs may provide a greater public health benefit.

## References

[1] Vinluan MH, Hofman J (2014). Transforming out-of-school time environments: the Y’s commitment to healthy eating and physical activity standards. Am J Health Promot.

[2] Beets MW, Weaver RG, Turner-McGrievy G, Moore JB, Webster C, Brazendale K, Chandler J, Khan M, Saunders R, Beighle A (2016). Are we there yet? Compliance with physical activity standards in YMCA afterschool programs. Child Obes.

[3] Beets MW, Huberty J, Beighle A, Moore JB, Webster C, Ajja R, Weaver G (2013). Impact of policy environment characteristics on physical activity and sedentary behaviors of children attending afterschool programs. Health Educ Behav.

[4] Beets MW, Shah R, Weaver RG, Huberty J, Beighle A, Moore JB (2015). Physical activity in after-school programs: comparison with physical activity policies. J Phys Act Health.

[5] Beets MW, Rooney L, Tilley F, Beighle A, Webster C (2010). Evaluation of policies to promote physical activity in afterschool programs: are we meeting current benchmarks?. Prev Med.

[6] Cradock AL, Barrett JL, Giles CM, Lee RM, Kenney EL, deBlois ME, Thayer JC, Gortmaker SL (2016). Promoting physical activity with the out of school nutrition and physical activity (OSNAP) initiative a cluster-randomized controlled trial. JAMA Pediatr.

[7] Dzewaltowski DA, Rosenkranz RR, Geller KS, Coleman KJ, Welk GJ, Hastmann TJ, Milliken GA (2010). HOP’N after-school project: an obesity prevention randomized controlled trial. Int J Behav Nutr Phys Act.

[8] Herrick H, Thompson H, Kinder J, Madsen KA (2012). Use of SPARK to promote after-school physical activity. J Sch Health.

[9] Kelder S, Hoelscher DM, Barroso CS, Walker JL, Cribb P, Hu S (2005). The CATCH kids club: a pilot after-school study for improving elementary students’ nutrition and physical activity. Public Health Nutr.

[10] Nigg CR, Geller K, Adams P, Hamada M, Hwang P, Chung R (2012). Successful dissemination of fun 5: a physical activity and nutrition program for children. Transl Behav Med.

[11] Sharpe EK, Forrester S, Mandigo J (2011). Engaging community providers to create more active after-school environments: results from the Ontario CATCH kids club implementation project. J Phys Act Health.

[12] Beets MW, Glenn Weaver R, Turner-McGrievy G, Saunders RP, Webster CA, Moore JB, Brazendale K, Chandler J. Evaluation of a statewide dissemination and implementation of physical activity intervention in afterschool programs: a nonrandomized trial. Transl Behav Med. 2017;7(4):690–01. 10.1007/s13142-017-0484-2 PMC5684072 28321706

[13] Des Jarlais DC, Lyles C, Crepaz N, Group T (2004). Improving the reporting quality of nonrandomized evaluations of behavioral and public health interventions: the TREND statement. Am J Public Health.

[14] Glasgow RE, Vogt TM, Boles SM (1999). Evaluating the public health impact of health promotion interventions: the RE-AIM framework. Am J Public Health.

[15] Gaglio B, Shoup JA, Glasgow RE (2013). The RE-AIM framework: a systematic review of use over time. Am J Public Health.

[16] Saunders R (2016). Implementation monitoring and process evaluation.

[17] Ogden CL, Carroll MD, Kit BK, Flegal KM (2012). Prevalence of obesity and trends in body mass index among US children and adolescents, 1999-2010. JAMA.

[18] Weaver RG, Beets MW, Webster C (2013). LET US play: maximizing children’s physical activity in physical education. Strategies: J Phys Sport Educ.

[19] Beets MW, Glenn Weaver R, Turner-McGrievy G, Huberty J, Ward DS, Freedman DA, Saunders R, Pate RR, Beighle A, Hutto B, Moore JB (2014). Making healthy eating and physical activity policy practice: the design and overview of a group randomized controlled trial in afterschool programs. Contemp Clin Trials.

[20] Beets MW, Weaver RG, Turner-McGrievy G, Huberty J, Ward DS, Pate RR, Freedman D, Hutto B, Moore JB, Beighle A (2015). Making policy practice in afterschool programs: a randomized controlled trial on physical activity changes. Am J Prev Med.

[21] Beets MW, Weaver RG, Turner-McGrievy G, Huberty J, Ward DS, Pate RR, Freedman D, Hutto B, Moore JB, Bottai M (2016). Physical activity outcomes in afterschool programs: a group randomized controlled trial. Prev Med.

[22] Beets MW, Okely A, Weaver RG, Webster C, Lubans DR, Brusseau T, Carson RL, Cliff DP (2016). The theory of expanded, extended, and enhanced opportunities for youth physical activity promotion. Int J Behav Nutr Phys Act.

[23] Brazendale K, Chandler JL, Beets MW, Weaver RG, Beighle A, Huberty JL, Moore JB (2015). Maximizing children’s physical activity using the LET US play principles. Prev Med.

[24] Beets MW, Beighle A, Bottai M, Rooney L, Tilley F (2012). Pedometer-determined step count guidelines for afterschool programs. J Phys Act Health.

[25] Beets MW, Huberty J, Beighle A (2012). Physical activity of children attending afterschool programs research- and practice-based implications. Am J Prev Med.

[26] Beets MW, Weaver RG, Moore JB, Turner-McGrievy G, Pate RR, Webster C, Beighle A (2014). From policy to practice: strategies to meet physical activity standards in YMCA afterschool programs. Am J Prev Med.

[27] Bailey RC, Olson J, Pepper SL, Porszaz J, Barstow TJ, Cooper DM (1995). The level and tempo of children’s physical activities: an observational study. Med Sci Sports Exerc.

[28] Baquet G, Stratton G, Van Praagh E, Berthoin S (2007). Improving physical activity assessment in prepubertal children with high-frequency accelerometry monitoring: a methodological issue. Prev Med.

[29] Vale S, Santos R, Silva P, Soares-Miranda L, Mota J (2009). Preschool children physical activity measurement: importance of epoch length choice. Pediatr Exerc Sci.

[30] Evenson KR, Catellier DJ, Gill K, Ondrak KS, McMurray RG (2008). Calibration of two objective measures of physical activity for children. J Sports Sci.

[31] Matthews CE, Chen KY, Freedson PS, Buchowski MS, Beech BM, Pate RR, Troiano RP (2008). Amount of time spent in sedentary behaviors in the United States, 2003-2004. Am J Epidemiol.

[32] Trost SG, Rosenkranz RR, Dzewaltowski D (2008). Physical activity levels among children attending after-school programs. Med Sci Sports Exerc.

[33] California Department of Education (2009). California after school physical activity guidelines.

[34] Hughey SM, Weaver RG, Saunders R, Webster C, Beets MW (2014). Process evaluation of an intervention to increase child activity levels in afterschool programs. Eval Program Plann.

[35] Weaver RG, Beets MW, Hutto B, Saunders RP, Moore JB, Turner-McGrievy G, Huberty JL, Ward DS, Pate RR, Beighle A, Freedman D (2015). Making healthy eating and physical activity policy practice: process evaluation of a group randomized controlled intervention in afterschool programs. Health Educ Res.

[36] Weaver RG, Moore JB, Huberty J, Freedman D, Turner-McGrievy B, Beighle A, Ward D, Pate R, Saunders R, Brazendale K (2016). Process evaluation of making HEPA policy practice: a group randomized trial. Health Promot Pract.

[37] Weaver RG, Beets MW, Webster C, Huberty J (2014). System for observing staff promotion of activity and nutrition (SOSPAN). J Phys Act Health.

[38] Metcalf B, Henley W, Wilkin T (2013). Effectiveness of intervention on physical activity of children: systematic review and meta-analysis of controlled trials with objectively measured outcomes (EarlyBird 54) (Reprint of 345, e5888, 2012). Br J Sports Med.

[39] Geraci M, Bottai M (2014). Linear quantile mixed models. Stat Comput.

[40] Cicchetti D, Toth SL (1995). A developmental psychopathology perspective on child abuse and neglect. J Am Acad Child Adolesc Psychiatry.

[41] Cicchetti D, Toth SL (1998). The development of depression in children and adolescents. Am Psychol.

[42] Afterschool Alliance (2014). America after 3 PM: a household survey on afterschool in America.

[43] Iversen CS, Nigg C, Titchenal CA (2011). The impact of an elementary after-school nutrition and physical activity program on children’s fruit and vegetable intake, physical activity, and body mass index: fun 5. Hawaii Med J.

[44] Nigg C, Battista J, Chang JA, Yamashita M, Chung R (2004). Physical activity outcomes of a pilot intervention using SPARK active recreation in elementary after-school programs. J Sport Exerc Psychol.

